# Dual functions of the *ZmCCT*-associated quantitative trait locus in flowering and stress responses under long-day conditions

**DOI:** 10.1186/s12870-016-0930-1

**Published:** 2016-11-03

**Authors:** Lixia Ku, Lei Tian, Huihui Su, Cuiling Wang, Xiaobo Wang, Liuji Wu, Yong Shi, Guohui Li, Zhiyong Wang, Huitao Wang, Xiaoheng Song, Dandan Dou, Zhaobin Ren, Yanhui Chen

**Affiliations:** 1 College of Agronomy, Synergetic Innovation Centre of Henan Grain Crops and National Key Laboratory of Wheat and Maize Crop Science, Henan Agricultural University, 95 Wenhua Road, Zhengzhou, 450002 China; 2 College of Agronomy, Henan University of Science and Technology, Luoyang, 471003 China

**Keywords:** Photoperiod, Flowering time, Stress tolerance, Co-expression network, Maize

## Abstract

**Background:**

Photoperiodism refers to the ability of plants to measure day length to determine the season. This ability enables plants to coordinate internal biological activities with external changes to ensure normal growth. However, the influence of the photoperiod on maize flowering and stress responses under long-day (LD) conditions has not been analyzed by comparative transcriptome sequencing. The *ZmCCT* gene was previously identified as a homolog of the rice photoperiod response regulator *Ghd7*, and associated with the major quantitative trait locus (QTL) responsible for *Gibberella* stalk rot resistance in maize. However, its regulatory mechanism has not been characterized.

**Results:**

We mapped the *ZmCCT*-associated QTL (*ZmCCT*-AQ), which is approximately 130 kb long and regulates photoperiod responses and resistance to *Gibberella* stalk rot and drought in maize. To investigate the effects of *ZmCCT*-AQ under LD conditions, the transcriptomes of the photoperiod-insensitive inbred line Huangzao4 (HZ4) and its near-isogenic line (HZ4-NIL) containing *ZmCCT*-AQ were sequenced. A set of genes identified by RNA-seq exhibited higher basal expression levels in HZ4-NIL than in HZ4. These genes were associated with responses to circadian rhythm changes and biotic and abiotic stresses. The differentially expressed genes in the introgressed regions of HZ4-NIL conferred higher drought and heat tolerance, and stronger disease resistance relative to HZ4. Co-expression analysis and the diurnal expression rhythms of genes related to stress responses suggested that *ZmCCT* and one of the circadian clock core genes, *ZmCCA1*, are important nodes linking the photoperiod to stress tolerance responses under LD conditions.

**Conclusion:**

Our study revealed that the photoperiod influences flowering and stress responses under LD conditions. Additionally, *ZmCCT* and *ZmCCA1* are important functional links between the circadian clock and stress tolerance. The establishment of this particular molecular link has uncovered a new relationship between plant photoperiodism and stress responses.

**Electronic supplementary material:**

The online version of this article (doi:10.1186/s12870-016-0930-1) contains supplementary material, which is available to authorized users.

## Background

Reproductive success, high yields and optimal regulation of floral transition processes and stress responses are critical for efficient crop production. All crop growth and developmental stages are influenced by various environmental factors, which can affect plant processes such as photosynthesis, respiration, germination, flowering, and stress tolerance. Day length (i.e., photoperiod) regulates plant responses to environmental signals and stresses [1], which enables plants to predict and respond to stress, as well as appropriately time their floral transition activities. Therefore, characterizing the photoperiod-related regulatory mechanisms underlying the timing of floral transition and stress tolerance is necessary to ensure reproductive success and increase crop yields.

The genetic architectures and molecular mechanisms associated with photoperiod-dependent flowering time regulatory pathways have been characterized in some species [2–7]. The best understood pathways include the photoperiod-based regulation of flowering time in the model dicot *Arabidopsis thaliana* and the model monocot rice (*Oryza sativa*). In contrast with the extensive genetic and molecular studies available regarding flowering time in *A. thaliana* and rice, there has been relatively little research on flowering time in maize (*Zea mays* ssp. *mays* L.), likely because of a lack of flowering time mutants. However, circadian clock core genes homologous to those in *A. thaliana* such as *CIRCADIAN CLOCK ASSOCIATED 1* (*CCA1*), *LATE ELONGATED HYPOCOTYL* (*LHY*), *TIMING OF CAB EXPRESSION 1a* (*TOC1a*), *TOC1b*, and *GIGANTEA* (*GI*), have been detected in the maize genome. Additionally, in maize, 10–23 % of these genes exhibit diurnal oscillations, which are key mRNA and protein features that have been largely conserved among various plant species [8–11].

Some important photoperiod-dependent maize genes have been characterized. Detailed studies of *ZmCCA1* and *ZmTOC1* have indicated that they are key components of the maize circadian clock [8, 12]. Additionally, a few candidate genes related to the maize photoperiod transduction pathway have been identified such as *CONSTANS 1* (*conz1*), *CCT* (*CO*, *CO-like*, *TOC1*), and *CENTRORADIALIS 8* (*ZCN8*) [13–15]. *CO1* and its upstream genes (i.e., *GI1a* and *GI1b*) exhibit diurnal expression patterns that are similar to those of their *A. thaliana* and rice homologs. *ZCN8* is a homolog of *Arabidopsis Flowering Locus T* (*FT*) as well as rice *HEADING DATE 3a* (*Hd3a*) and *RICE FLOWERING LOCUS T1* (*RFT1*), and is considered to function as a florigen in maize [13]. The diurnal oscillation of maize *ZCN8* expression is upregulated in the leaves of photoperiod-sensitive tropical lines when exposed to long-day (LD) conditions. In contrast, a weak diurnal pattern is observed in day-neutral temperate lines. Downregulation of *ZCN8* expression via artificial microRNA leads to late flowering. *ZCN8* was mapped downstream of *INDETERMINATE 1* (*ID1*) and upstream of *DELAYED FLOWERING 1* (*DLF1*) [13]. *ZmCCT* is the homolog of the rice photoperiod response regulator *Ghd7*, which was identified by nested association mapping of natural variants. Association mapping panels revealed that it has an essential role in maize photoperiod responses [8, 15, 16]. Under LD conditions, teosinte *ZmCCT* alleles are continuously upregulated and confer delayed flowering unlike the corresponding maize alleles [8].

There is accumulating evidence that the photoperiod is important for plant responses to abiotic and biotic stresses [17–22], including drought, heat, or disease, which cause extensive agricultural losses worldwide. Furthermore, the significant changes in temperatures resulting from global warming have disrupted plant growth and reduced crop yields [23, 24]. Therefore, generating crops with enhanced tolerance to changes in field conditions offers an approach to decrease yield losses, improve growth, and ensure a sufficient food supply for the continuously growing world population [24]. Jones et al. [20] revealed that the major plant immune mechanism against biotrophic pathogens involves resistance (*R*)-gene-mediated defense. Wang et al. [21] identified novel genes responsible for *R*-gene-mediated resistance to downy mildew in *A. thaliana*, as well as their control via the circadian regulator *CCA1*. Numerical clustering based on the phenotypic features of mutants in these genes indicated that programmed cell death is the predominant contributor to resistance. These new defense genes were observed to be under circadian regulation by *CCA1*, thereby enabling plants to ‘anticipate’ infection at dawn, which is the optimal time for the pathogen to disperse its spores. Min et al. [22] revealed that the expression of *AtCO-like 4* (*AtCOL4*) is strongly stimulated by abscisic acid, as well as osmotic and salt stresses, which indicated *AtCOL4* is an essential regulator of tolerance to abiotic stresses in plants.

The molecular mechanisms underlying the regulation of photoperiod-dependent flowering time in maize remain elusive and, importantly, the link between photoperiodic pathway genes and plant stress tolerance has not been well established. Here, we used the photoperiod-sensitive inbred line HZ4-NIL and the photoperiod-insensitive inbred line HZ4 to investigate the transcriptomic changes occurring under LD conditions. Our objective was to clarify the role of the *ZmCCT* **-**associated quantitative trait locus (QTL) in flowering and stress responses. This research should extend our understanding of the genetic mechanisms underlying photoperiod-dependent flowering time and stress tolerance in maize.

## Methods

### Plant materials and fine mapping of qDPS10

The maize inbred lines CML288 (donor parent; tropical LD photoperiod-sensitive) acquired from the National Maize and Wheat Improvement Center in Mexico, and Huangzao 4 (recurrent parent; temperate photoperiod-insensitive), a representative of the Chinese Tangsipingtou heterotic group, were selected to develop various mapping populations, including multiple backcross populations (BC_1_F_1_, BC_2_F_1_, BC_3_F_1_, BC_4_F_2_, BC_5_F_1_, BC_6_F_1_, and BC_7_F_1_). All mapping populations were grown at the experimental farm of Henan Agricultural University (Zhengzhou, Henan, China). A schematic diagram illustrating the development of the near-isogenic lines of Huangzao 4 (HZ4-NIL) has been published [16].

To develop molecular markers for fine mapping, bacterial artificial chromosome sequences of the B73 genome in the region flanked by umc1873 and umc1053 on chromosome 10 were obtained from the maize Genetics and Genomics Database (MaizeGDB; http://gbrowse.maizegdb.org/gb2/gbrowse/maize_v2). Simple sequence repeats (SSRs) were identified using the SSR Hunter Software [25]. Primers were designed using the Primer Premier 5.0 software (Premier Biosoft International, Palo Alto, CA, USA) to generate PCR products that were <300 bp. The primer sequences used in this study are listed in Additional file 1: Table S1.

### Experimental treatments

The HZ4 and HZ4-NIL plants were grown in growth chambers (2.8 × 5.6 × 8.2 m) under LD conditions (15-h light/9-h dark, 25 °C), with a light intensity of 100 μmol m^−2^ s^−1^ in Zhengzhou, China, in the spring of 2012. We defined three developmental stages for RNA-seq analysis (i.e., vegetative stage: 3-fully expanded leaf period, the transition from vegetative to reproductive growth: 4- and 5-fully expanded leaf periods, reproductive stage: 6-fully expanded leaf period for the photoperiod-insensitive inbred line HZ4; vegetative stage: 3-fully expanded leaf period, the transition from vegetative to reproductive growth: 5- and 6-fully expanded leaf periods; and reproductive stage: 7-fully expanded leaf period for the photoperiod-sensitive inbred line HZ4-NIL). We compared the differentially expressed genes (DEGs) between the two inbred lines at each stage (i.e., 3-fully expanded leaf period in HZ4/3-fully expanded leaf period in HZ4-NIL; 4-fully expanded leaf period in HZ4/5-fully expanded leaf period in HZ4-NIL; 5-fully expanded leaf period in HZ4/6-fully expanded leaf period in HZ4-NIL; 6-fully expanded leaf period in HZ4/7-fully expanded leaf period in HZ4-NIL). For downstream analysis by RNA-seq and shoot apical meristem (SAM) analysis, HZ4 seedlings were harvested at the 3-, 4-, 5-, and 6-fully expanded leaf stages, while HZ4-NIL plants were collected at the 3-, 5-, 6-, and 7-fully expanded leaf stages. At each stage, 19 seedlings were collected. Five seedlings with equal amounts of leaves and other tissues were pooled for RNA-seq analysis, while another five plants were used for SAM analysis. Additionally, three seedlings were combined to analyze gene expression via the quantitative reverse transcription polymerase chain reaction (qRT-PCR). Three independent biological replicates were used for the gene expression validation.

### Shoot apical meristem analysis

We analyzed the SAMs of five symmetrical plants from each inbred line grown under LD conditions at each developmental stage as previously described [25]. Briefly, the maize stem tips were fixed in FAA and extensively rinsed in 70 % ethanol. The SAMs were then peeled off under dissecting optics. Next, the maize SAMs were stained using 20 μg mL^−1^ Hoechst 33258 (TaKaRa Biotechnology Company, Dalian, China) at 25 °C for 24 h in the dark. Finally, the morphology of the maize SAMs was examined under a laser scanning confocal microscope (Leica TCS-SP2) [26].

### Phenotype identification during stress under LD conditions

#### Plant materials and culture

HZ4 and HZ4-NIL seeds were surface sterilized in 10 % H_2_O_2_ for 20 min, rinsed in distilled water, and then allowed to germinate for 2 days between two layers of dampened filter paper at 28 °C in darkness. Seedlings (1–2-cm tall) were transferred to vermiculite and allowed to grow under a 28 °C, 15-h light/22 °C, 9-h dark cycle. Seedlings (2-fully expanded leaf stage) of uniform height were transferred to 2-L pots containing full-strength Hoagland’s nutrient solution [27]. The seedlings were grown under LD conditions (15 h light/9 h dark) in a controlled-temperature culture room at 22 °C and a 60 % relative humidity. The nutrient solution was replaced every 2 days. Seedlings with three leaves were used for abiotic stress treatments.

#### Stress treatments

For artificial inoculation in the field, maize kernels were sterilized as previously described [28] and incubated with an agar slab containing *Fusarium graminearum* at 25 °C in complete darkness for 15 days. Thoroughly mixed infected maize kernels were used to inoculate plants on the silking date by burying the kernels (approximately 70 g) in the ground 5–10-cm away from the stem. To promote fungal growth and infection, the field was irrigated to increase soil moisture levels. Plants were examined for stalk rot symptoms according to an established method [28]. Heat stress was induced by incubating plants (3-fully expanded leaf stage) at 40 °C for 4 days. For drought treatment, 20 % polyethylene glycol was added to the nutrient solution for 1 day. Total RNA was extracted from the seedlings (Additional file 1: Table S1). Control seedlings were grown under the same conditions but without the polyethylene glycol treatment.

The relative water contents (RWCs) of HZ4 and HZ4-NIL were analyzed to identify phenotypic differences under drought and heat stress conditions. Detached leaves were weighed, saturated with water for 24 h and weighed again, and then dried for 48 h and weighed a third time. The RWC was calculated using the following formula: RWC (%) = [(FM − DM)/(TM − DM)] × 100, where FM, DM, and TM refer to the fresh, dry, and turgid masses of the tissue, respectively [29]

### RNA extraction, RNA-seq library construction, and sequencing

Five leaves or SAM samples were harvested from plants grown under LD conditions. Samples were collected at each new fully expanded leaf stage (maize leaves were defined as fully expanded when the new leaf’s sheath just appeared from the lower leaf’s sheath, or the new leaf’s ligule overlapped the lower leaf, and the whole leaf blade fully extended from the lower leaf) and pooled for each genotype (HZ4 and HZ4-NIL). All samples were flash-frozen in liquid nitrogen and then stored at −80 °C. We used TRIzol reagent (Invitrogen, Carlsbad, CA, USA) to extract total RNA, which was treated with DNase I and magnetic oligo (dT) beads. cDNA was synthesized using random hexamers and SuperScript II Reverse Transcriptase (Life Technologies, Ontario, Canada). Libraries were constructed and sequenced as previously described [30]. The cDNA libraries were sequenced using a sequence-by-synthesis technique on the HiSeq 2000 platform (Illumina) at the Beijing Genomics Institute (Beijing, China).

### Transcriptome data analysis

An in-house Perl script was used to remove the paired-end reads containing >5 % ambiguous residues (Ns) and reads of more than 10 % bases with a Phred score <20. The remaining reads were considered “clean reads” [31]. The high-quality pair-end reads from each sample were mapped to the maize cv. B73 RefGen_V3 genomic DNA sequence using the TopHat software [32]﻿. The reads were then assembled using Cufflinks (version 2.0.2) [33] to discover novel transcripts (using the parameters: –g –b –u –o (–g/–GTF-guide: use reference transcript annotation to guide assembly; –b/–frag-bias-correct: use bias correction-reference FASTA required; –u/–multi-read-correct: use the ‘rescue method’ for multi-reads; –o/–output-dir: write all output files to this directory) [34–36]. The default parameters of Cuffdiff were used to calculate the expression level changes and the associated q-values (false discovery rate adjusted *P*-values) of each gene. Finally, the genes were further classified as significantly differentially expressed when the following three conditions were fulfilled: q ≤ 0.05, |fold change| ≥ 1.5, and the FPKM-normalized expression level of at least one of the two samples was higher than the 25th percentile [37, 38].

Gene function annotations were performed using Gene Ontology (GO) (http://www.geneontology.org/) and WEGO (http://wego.genomics.org.cn/). AgriGO was used for GO enrichment analysis of all identified DEGs in the two genotypes. Additionally, the enriched GO categories (Reference Genome Group of the Gene Ontology, 2009) among the common DEGs in both organs were detected with the Cytoscape (version 3.0.2) plugin ClueGO + Cluepedia (version 2.1.3) [39, 40]. The GO categories searched included biological processes and Kyoto Encyclopedia of Genes and Genomes (KEGG) pathways.

We used the Short Time-series Expression Miner (STEM) software package [41] to identify genes that were up- or downregulated at specific developmental stages based on the time-course expression data. The STEM clustering method (http://www.cs.cmu.edu/~jernst/stem/) was used to evaluate the DEGs of leaves and SAMs in HZ4 and HZ4-NIL plants. This clustering method initially defines a set of distinct and representative model temporal expression profiles that correspond to changes in the expression of each gene over time, independent of the data. All model profiles started at 0, and model profiles were maintained between pairs of time points. An increase or decrease in expression was represented by an integral number of units. Each DEG was assigned to the model profile to which its time series most closely matched based on the correlation coefficient. The number of HZ4 and/or HZ4-NIL DEGs assigned to each model profile was then determined. Additionally, the number of DEGs expected to be assigned to each profile by chance was calculated by randomly performing permutations of the original time point values, and then renormalizing the expression values and assigning them to the most closely matched model profiles. The procedure was repeated using a large number of permutations. The average number from all permutations was used as the estimate of the expected number of DEGs for HZ4 and/or HZ4-NIL assigned to each profile. The significance of the number of genes assigned to each profile versus the expected number was then calculated to determine whether the profile identified more or fewer HZ4 and/or HZ4-NIL DEGs than expected by chance.

Pearson correlation coefficients were calculated for all genes related to circadian rhythms and stress responses detected in the HZ4 and HZ4-NIL leaves and SAMs [cutoff values at adjusted *P* < 1.0 × 10^−8^ (BH method)]. We used the igraph R package (version 0.6–3) to construct a gene co-expression network. To confirm that the resulting network was reasonable for a biological network, we used the methods previously described by Yasunori et al. [42]. Cytoscape (v3.0.2) was used for network visualization and enrichment with various data (i.e., differential expression data).

### Analysis of *cis*-acting elements and diurnal rhythms for the differentially expressed genes identified in the co-expression network

*Cis*-acting regulatory elements in the promoter regions [the 3,000-bp region upstream of ATG (start codon)] of the DEGs were identified in the co-expression network using the PLACE [43] and PlantCARE [44] databases. To investigate the diurnal rhythms under LD conditions, HZ4 and HZ4-NIL leaves and shoot apices were collected at the new fully expanded 5-fully expanded leaf stage. Samples were harvested from both genotypes every 2 h over a 48-h period. Three biological replicates were used for each experiment.

### Validation of DEG status using real-time RT-PCR

To validate the cDNA sequencing results, leaves and SAMs from three seedlings (three biological replicates per sample) were pooled and RNA was extracted as described above. Total RNA was treated with DNase I, and cDNA was synthesized using the Easy-Script First-Strand cDNA synthesis SuperMix (Transgen, Beijing, China). A qRT-PCR assay, as described by Wang et al. [12], was conducted to verify a subset of DEGs. Gene sequences were downloaded from the Gramene maize database (http://ensembl.gramene.org/Zea_mays/Location). The Primer 3.0 software (http://primer3.ut.ee/) was used to design the primers (Additional file 2: Table S5). A total of 39 maize genes from various functional categories were analyzed by qRT-PCR. Reactions were completed in 25-μL volumes using a SYBR Green PCR Master Mix kit (Applied Biosystems, Foster City, CA, USA) and a Light Cycler® 480II Sequence Detection System. Relative gene expression levels were calculated using the 2^−ΔΔCt^ method [45]. The *l8S rRNA* gene was used as an endogenous reference, and all analyses were conducted with three technical and biological replicates.

## Results

### Fine mapping of a major quantitative trait locus for photoperiod sensitivity and biotic stress responses

**Fig. 1:**
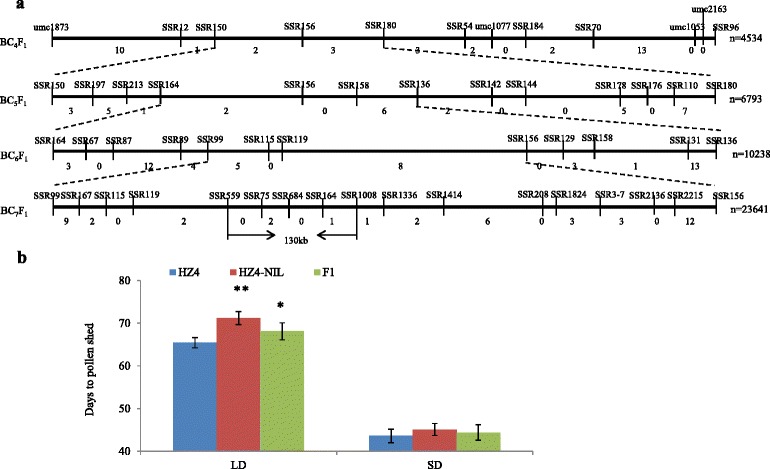
Sequential fine mapping of qDPS10 and flowering time in HZ4, HZ4-NIL and the F1 (HZ4 × HZ4-NIL). **a** Location of fine-mapped regions in the chromosome 10. The qDPS10 locus was primarily mapped between SSR markers SSR150 and SSR180 in chromosome 10, and fine mapped between markers SSR559 and SSR1008 with the physical distance of 130 kb. **c** Days to pollen shed under long-day (LD; Zhengzhou, Henan) and short-day (Sanya Hainan) conditions

We previously mapped qDPS10 on chromosome 10 between the markers umc1873 and umc1053 for days to pollen shed (DPS) in LD environments [12]. To fine-map qDPS10, we generated mapping populations derived from a cross between the temperate photoperiod-insensitive inbred line HZ4 (the recurrent parent) and the tropical photoperiod-sensitive inbred line CML288 (the donor parent). The populations included a BC_4_F_2_ with 4,534 plants, a BC_5_F_1_ with 6,793 plants, a BC_6_F_1_ with 9,275 plants, and a BC_7_F_1_ with 21,173 plants. Screening with molecular markers (Additional file 1: Table S1) mapped qDPS10 to a 130-kb region between markers SSR559 and SSR1008 (Fig. 1a). Within this region, four predicted genes or open reading frames were identified. According to a bioinformatics analysis, these sequences encoded a pseudogene, a CCT domain transcription factor, and two transposable elements. The CCT domain gene (GRMZM2G381691) in qDPS10 was considered a candidate gene for photoperiod sensitivity. The gene was previously named *ZmCCT*, and fine-mapping showed allelic variants that possibly modulated flowering time [15, 16]. Furthermore, the molecular mechanism of *ZmCCT* was previously verified by maize genetic transformation and association analysis [15]. Additionally, Yang et al. [28] identified a QTL spanning the *ZmCCT* locus for resistance to *Gibberella* stalk rot in maize using a mapping population that was derived from a cross between varieties “1145” (donor parent, completely resistant) and “Y331” (recurrent parent, highly susceptible) by fine-mapping.

### Phenotypic variation in flowering time and stress responses under long-day conditions

**Fig. 2:**
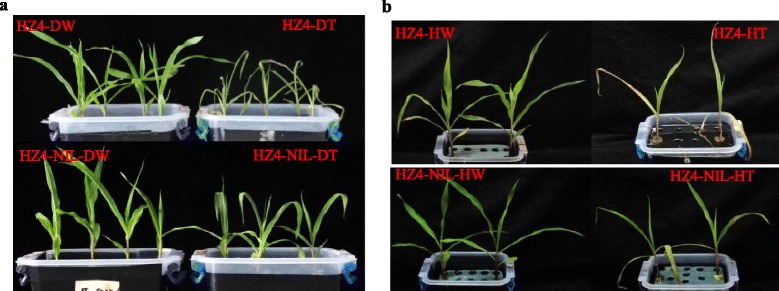
Phenotypic variations in HZ4 and HZ4-NIL responses to stress under long-day conditions. **a** Phenotypes under drought treatment. D: drought conditions, W: control samples, T: treated samples. **b** Phenotypes under high temperature. H: High temperature treatment

There were no significant differences between HZ4 and HZ4-NIL in flowering time under short-day conditions (9-h light/15-h dark, 25 °C, in Zhengzhou, China, in the spring of 2012), whereas HZ4 plants flowered 6 days earlier than HZ4-NIL plants under LD conditions (*P* < 0.01; Fig. 1b). The HZ4 and HZ4-NIL plants also differed in terms of drought tolerance, heat tolerance, and disease reactions under LD conditions (Fig. 2, Additional file 3: Figure S1a). To investigate the physiological difference in the drought tolerance of two genotypes, the RWC was determined for leaves harvested from seedlings (3-fully expanded leaf stage) exposed to drought and heat stresses. The RWC in HZ4-NIL (68.7 %) leaves was significantly higher than that in HZ4 (48.6 %) leaves after 1 day of drought stress (*P* < 0.01). The RWC in HZ4-NIL (61.37 %) leaves was also significantly higher than that in HZ4 (40.18 %) leaves after 4 days of heat stress (*P* < 0.01). Regarding disease reactions under LD conditions, approximately 78 % of the HZ4-NIL plants were highly resistant to *Gibberella* stalk rot with only minor symptoms observed in the field. In contrast, approximately 70 % of HZ4 plants were severely infected and exhibited severe stalk rot symptoms (Additional file 3: Figure S1a). These results indicated that LD conditions not only affected flowering time, but also responses to stresses such as drought and high-temperature, and disease resistance in HZ4-NIL plants.

To investigate the potential difference between HZ4 and HZ4-NIL plants in terms of photoperiod-dependent floral transitions, we analyzed individual SAMs harvested from plants (3- to 7-fully expanded leaf stages) grown under LD conditions. Morphologically, the SAMs were similar between the two genotypes at the 3-fully expanded leaf stage. However, at the 4- to 7-fully expanded leaf stages, the HZ4 SAM appeared similar to the HZ4-NIL SAM from the previous leaf stage (Additional file 3: Figure S1b). These results indicated that the floral transition occurred one leaf period earlier in HZ4 than in HZ4-NIL.

### Transcriptome sequencing and global gene expression profiles under long-day conditions

Using the Illumina SBS (sequence by synthesis) technique on a HiSeq 2000 (Illumina) sequencing platform, between 25 and 28 million 100-nt reads were generated for each RNA sample (Additional file 3: Figure S2a, b). Approximately 67.23–74.80 % of the reads from each sample were mapped to the maize genome using Bowtie, with no more than five misaligned positions. Of the mapped reads, approximately 64 % were mapped to a unique position (Additional file 3: Figure S2c and d). Therefore, our RNA-seq data appeared to adequately represent the complexity of the gene expression profiles within the four developmental periods.

To characterize the relationships among various samples, we conducted a Pearson correlation coefficient (PCC) analysis of the sequenced libraries representing the three samples. Additional file 3: Figure S3 shows that the gene expression profiles in leaves and SAMs were clustered into two groups, and each of the analyzed comparison periods in these two genotypes (Additional file 3: Figure S1b) showed relatively high similarities, supporting our previous observations and reflecting the similar genetic backgrounds.

Based on a qRT-PCR analysis of 20 candidate genes, we established 20 reads as a cutoff to determine the number of expressed genes across the 16 samples. By this criterion, a total of 27,542 genes were expressed in leaves and 29,774 genes were identified as expressed in SAMs (Additional file 3: Figure S4a). Approximately 431, 215, 795 and 503 genes were expressed specifically in LHZ4, LHZ4-NIL, SHZ4, and SHZ4-NIL, respectively. Furthermore, 24,798 genes (83.29 %) produced transcripts that were detected in all samples (Additional file 3: Figure S4a). We detected numerous genes that were differentially expressed between HZ4 and HZ4-NIL specifically in the SAMs/leaves (507/357) at the 3-fully expanded leaf stage (498/370). In contrast, 661/512, 682/734, and 726/638 DEGs were detected between HZ4 and HZ4-NIL in the SAMs/leaves during the other developmental stages (Additional file 3: Figure S4b). These results indicated that the transcriptomes generated in the four examined developmental stages were highly complex.

### Identification of temporally up- and downregulated differentially expressed genes under long-day conditions

**Fig. 3:**
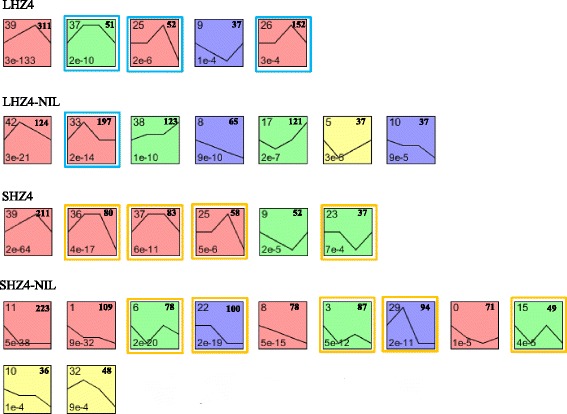
Expression profiles and clusters of differentially expressed genes obtained from Short Time-series Expression Miner clustering. The upper numbers indicate clusters or profiles. Clusters are arranged according to the number of genes, whereas profiles are classified according to significance. Significantly different profiles are represented by different background colors

Four and five general temporal gene expression patterns in leaves and SAMs, respectively, were determined by STEM analysis to be significantly different in HZ4 during the four stages (*P* < 0.001, Fig. 3). The genes and log fold-changes for the significantly enriched profiles are presented in Additional file 4: Table S6. Similar expression profiles were detected for leaves and SAMs (i.e., profiles 39, 37, 25, and 9 in leaves, and profiles 8 and 10 in SAMs; Fig. 3). These results indicated that most DEGs in the same genotype exhibited similar expression patterns regardless of tissue (i.e., leaves or SAMs). Although the two genotypes did not generate exactly the same profiles in the same tissues, 42.29 % (profiles 37, 25, and 26) and 27.98 % (profile 33) of the identified DEGs in the significant model profiles showed altered gene expression patterns during the transition stage (i.e., 4LHZ4 to 5LHZ4 and 5HZ4-NIL to 6HZ4-NIL). However, 42.42 % (profiles 36, 37, 25, and 23) and 36.90 % (profiles 6, 22, 3, and 29) showed similar SAM expression profiles during the transition stage (Fig. 3). Several significant model profiles also revealed a single change point in leaves (profiles 39, 9, 42, and 5) and SAMs (profiles 39 and 9) in HZ4 and HZ4-NIL (Fig. 3). These results indicated that the gene expression patterns of the photoperiod-insensitive inbred line HZ4 differed from those of the photoperiod-sensitive inbred line HZ4-NIL. Furthermore, only some of the leaf and SAM gene expression patterns were the same for HZ4 and HZ4-NIL under LD conditions. This enabled the identification of DEGs in different germplasm and tissues. These findings provided evidence that the *ZmCCT*-associated QTL (*ZmCCT*-AQ) caused the gene expression levels in HZ4-NIL to differ from those in HZ4 at the same stage (Additional file 3: Figure S5b and c), leading to specific gene expression patterns during development. Finally, our results confirmed that the genetic background of *ZmCCT*-AQ was highly complex and that clarifying the mechanism underlying the effects of *ZmCCT*-AQ was warranted.

**Fig. 4:**
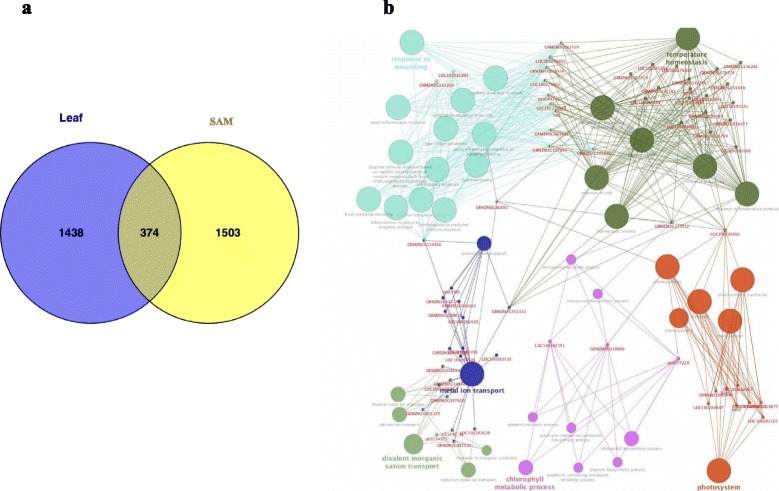
Expression and functional analysis of DEGs from HZ4-NIL compared with HZ4 in all leaf periods under long-day conditions (**a**) Venn diagram of DEGs identified in different organs (leaf and shoot apex). (**b**) GO enrichment analysis of common DEGs identified in leaves and SAMs. The DEGs were analyzed using the Cytoscape plug-in ClueGO + Cluepedia to identify statistically enriched GO categories compared with the ClueGO maize reference genome. Nodes represent a specific GO term and are grouped based on the similarity of their associated genes. Each node represents a single GO term and is color-coded based on enrichment significance. Node size indicates the number of genes mapped to each term

The backcross introgression strategy has been widely used for crop improvement. Introgressions integrate the genetic background of the recurrent parent into the progeny, which can lead to unique gene expression changes. To examine the effect of introgression on the transcriptome of HZ4-NIL under LD treatment, the genome-wide gene expression patterns of HZ4 and HZ4-NIL under LD treatment were compared. The results indicated that 636/588 and 1,230/1496 genes from leaves/SAMs were up- and downregulated, respectively, in HZ4-NIL relative to the levels in HZ4 in all leaf stages (Additional file 3: Figure S4c). Only a small proportion of DEGs (374 up-/downregulated genes in both leaves and SAMs) was associated with the introgressed regions (Fig. 4a).

Stringent GO term enrichment analysis of the DEGs under LD conditions revealed that key biological processes (e.g., metabolic processes, oxidation reduction, carbohydrate metabolic processes, and responses to external stimulus) and molecular functions (e.g., catalytic activity, oxidoreductase activity, and electron carrier activity) were significantly enriched (Additional file 5: Table S2). Additionally, GO analysis indicated that the common DEGs from leaves and SAMs in the four leaf development stages could be classified into the following three groups: cellular components (including ‘cell’, ‘cell part’, and ‘organelle’), molecular functions (such as binding and catalytic activity), and biological processes (including metabolism, cellular processes, biological regulation, pigmentation, and responses to stimulus) (Additional file 3: Figure S5). Finally, a biological development and KEGG pathway network consisting of common DEGs was constructed using the Cytoscape (v3.0.2) plugin ClueGO + Cluepedia (v2.1.3). The network included 41 GO and KEGG terms, 63 connected gene nodes, and 442 edges (Fig. 6). Furthermore, the network was divided into approximately six parts (mainly comprising responses to wounding, temperature homeostasis, and the photosystem) based on biological process and pathway information (Fig. 4b). Numerous genes were associated with more than two function or pathway terms, such as GRMZM2G381691, GRMZM2G352132, GRMZM2G177412, GRMZM2G314660, and LOC10028186 (Fig. 4b). In particular, GRMZM2G381691 and GRMZM2G352132 were related to temperature homeostasis, the photosystem, and metal ion transport. These results indicated that DEGs identified from RNA-seq data were possibly regulated by the photoperiod, but were also associated with defense responses. Additionally, introgression contributed to photoperiod sensitivity and the expression of stress-related phenotypes in HZ4-NIL plants.

### Gene co-expression networks in response to long-day treatment

**Fig. 5:**
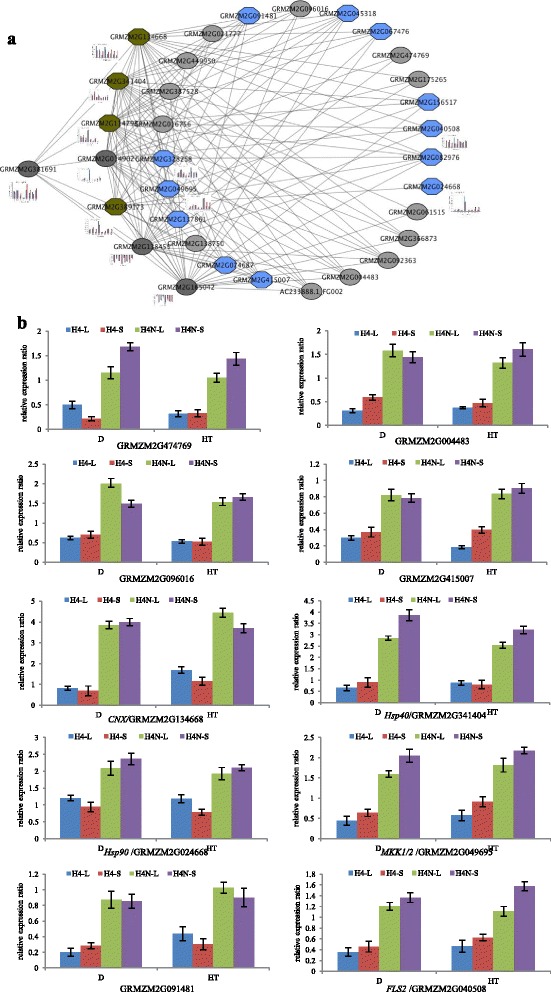
(**a**) Gene co-expression network. The co-expression network was generated by assigning edges using Pearson’s correlation coefficient for the common DEGs from both leaves and SAMs under long-day conditions. Nodes represent gene names and an edge between two nodes (genes) represents co-expression of the genes. The colours of the nodes represent different functional profiles. Light grey nodes represent photoperiod association, while blue nodes represent biotic stress association. Similarly, dark brown nodes represent abiotic stress association. (**b**) Relative expression ratios between HZ4 and its NIL for some co-expressed genes in the network in leaves (L) and SAMs (S) after drought (D) and high temperature (HT) treatment under long-day conditions. The relative expression ratio = (relative expression of NIL − relative expression of HZ4)/relative expression of HZ4

To compare the genetic networks of HZ4-NIL relative and HZ4 under LD treatment, the common DEGs from both leaves and SAMs belonging to the above functional categories were used in co-expression network analysis. Thirty-three of the DEGs were determined to be co-regulated, and formed a complex network (Fig. 5a); all genes in this network were validated by qRT-PCR (Fig. 5a, Additional file 6: Figure S6). We found that the gene expression profiles of these DEGs identified using qPCR revealed similar variation trends to the RNA-seq samples, indicating that the RNA-seq analysis was well suited for analysis of maize transcriptomic responses to long days. The genes in the network were then separated into three profiles based on their putative functions (Additional file 7: Table S3). Profile A genes were involved in circadian rhythm pathways. Fourteen genes were related to transcription regulation, including five C2C2-CO zinc finger proteins, two C2C2-Dof zinc finger proteins, three basic Helix-Loop-Helix (bHLH) family proteins, three MYB-related family proteins, and one CCAAT-HAP2 family protein. Three genes encoded enzyme proteins, including two synthetases and one peroxiredoxin. Profile B was enriched in genes associated with abiotic stress signal transduction, including six chaperone proteins and one ubiquitin-conjugating enzyme protein. Profile C genes were mainly involved in biotic stress responses, and included two chaperone proteins, one channel protein, three protein kinases, one MYB-like transcription factor, and one Derlin family protein.

**Fig. 6:**
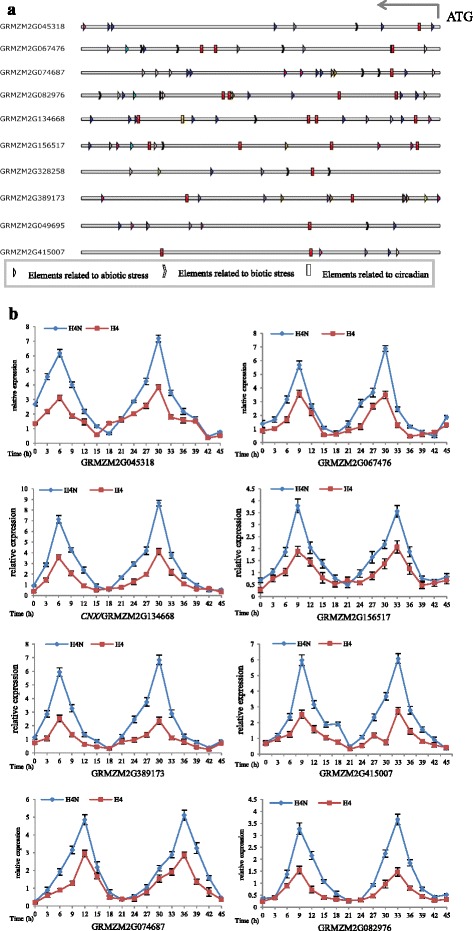
*Cis*-acting regulatory elements and expression of co-expressed genes related to stress. (**a**) *Cis*-acting regulatory elements identified in some DEG promoter regions (the 3000-bp region upstream of ATG (start codon)) using the PLACE and PlantCARE databases. Different colors indicate the various cis-elements related to the three stress responses. (**b**) Diurnal rhythms of expression for coexpressed genes related to stress with elements related to circadian rhythms from the networks in HZ4 and its NIL in long-day conditions

To identify the links between circadian rhythm and stress responses, the promoter regions of the genes associated with stress responses were analyzed using the PLACE and PlantCARE databases. Significant enrichment of the “evening element” was observed, with 62.5 % of the genes involved in abiotic and biotic stress responses containing this element in their promoters (Fig. 6a). Some elements in the −3,000-bp promoter region upstream of the start codon were predicted to be related to responses to light and hormones (Additional file 8: Table S4). Further analyses revealed that 8 of 10 co-expressed stress response-related genes containing the evening element exhibited rhythmic expression patterns (Fig. 6b).

## Discussion

The circadian rhythm is one of the most important biological rhythms that help plants adapt to the external world. The diurnal light/dark period is an important environmental factor that induces flower formation. Flowering time, which reflects the transition from vegetative to reproductive growth in plants, is also one of the major traits associated with maturation and adaptation. Genetic regulatory networks have been generated that indicate flowering time in *A. thaliana* is induced by circadian rhythms, and are often presented in graphical form [46–48]. However, our understanding of the role of circadian rhythms in plant stress responses is limited. We mapped the *ZmCCT*-associated DNA fragment (*ZmCCT*-AF) comprising a nearly 130-kb QTL from HZ4-NIL that regulates photoperiod responses and resistance to *Gibberella* stalk rot and drought in maize. To investigate the transcriptomic influence of this fragment under LD conditions, the transcriptomes of HZ4 and HZ4-NIL containing *ZmCCT*-AF were sequenced. A set of genes with higher basal expression levels in HZ4-NIL than in HZ4 was revealed to function in circadian responses, as well as in some biotic and abiotic stress tolerance responses. The DEGs within the introgressed regions of HZ4-NIL conferred higher drought and heat tolerance and stronger disease resistance relative to the recurrent parent HZ4. Our co-expression analysis and the diurnal rhythms of stress response-related genes suggest that *ZmCCT* and one of the circadian clock core genes, *ZmCCA1*, are important nodes linking photoperiod with stress tolerance responses under LD conditions.

### Effect of introgression on the transcriptome regarding flowering time and stress responses under LD conditions

Hung et al. [15] and Yang et al. [16] reported that *ZmCCT*, which encodes a CCT domain-containing protein, is the most important photoperiod-dependent regulator of flowering time, and that the upregulation of *ZmCCT* results in a delayed flowering time. Thus, some of the DEGs in the introgressed regions of HZ4-NIL directly affect flowering time. Additionally, the DEGs in these introgressed regions also directly influence drought and heat tolerance, and alter the disease reaction phenotype (Fig. 2, Additional file 3: Figure S1a). In this study, a set of genes was differentially expressed between HZ4 and HZ4-NIL that included genes related to responses to drought, temperature, and biotic stress (Fig. 5b). Further analyses revealed that some genes associated with responses to drought and heat stress were more highly expressed in HZ4-NIL than in HZ4 under LD conditions (Fig. 5b). These genes expression changes were consistent with the phenotypes of HZ4 and HZ4-NIL plants in response to stress under LD conditions. Additionally, the stress-related genes carrying the evening element exhibited circadian expression patterns, indicating that the photoperiod regulates flowering time as well as stress responses under LD conditions (Fig. 6b). Wang et al. [21] also identified a key relationship between circadian rhythm and plant immunity in *A. thaliana*.

### A complex gene expression regulatory network affects flowering time and stress tolerance

Several QTL mapping studies have indicated that photoperiod-dependent flowering time in maize involves a complex genetic architecture [3, 49, 50]. In contrast to *A. thaliana*, in which at least 100 flowering time genes have been characterized [46, 51], only a few maize flowering time QTLs and mutants have been isolated. In the present study, we observed that *ZmCCT* (GRMZM2G381691) had the biggest influence on *ZmCCA1* (GRMZM2G014902), *ZmPIL5* (GRMZM2G165042), and *ZmCDF1* (GRMZM2G138455) (Fig. 5a). In *A. thaliana*, *CCA1* exhibits robust circadian oscillations at both the RNA and protein levels. *CCA1* directly suppresses *TOC1* expression by binding to its promoter [52, 53]. The higher *ZmCCAl* expression levels in HZ4-NIL than in HZ4 decreased the expression levels of downstream genes of *ZmCCA1* (i.e., *GI*, *CO* and *FT*) and resulted in delayed flowering in this study. This result is consistent with the finding of Wang et al. [11], who determined that *ZmCCA1* overexpression in *A. thaliana* reduces the expression levels of downstream genes, including *AtGI*, *AtCO* and *AtFT*, resulting in longer hypocotyls and delayed flowering [11]. *PIL5* encodes a basic helix-loop-helix transcription factor, and preferentially interacts with the Pfr forms of *PhyA* and *PhyB. CDF1* is an important repressor of *CO* and *FT* expression in the morning [54–56]. Our findings indicate that *ZmCCT* delays flowering time in HZ4-NIL plants under LD conditions by downregulating an upstream gene (*PIL5*), and upregulating the circadian clock gene *CCA1* and the downstream gene *CDF1* (Additional file 7: Table S3).

Stress tolerance is considered a complex trait involving several genes. Therefore, deciphering the molecular mechanisms underlying stress tolerance in plants is a challenging task. In the present study, we observed a key functional link between the photoperiod and stress tolerance in maize. *ZmCCT* (GRMZM2G381691) had the biggest influence on protein disulfide isomerases (*PDI*s; GRMZM2G389173), *BiP* (*Hsp70*; GRMZM2G114793), *Hsp40* (GRMZM2G341404), and calnexin (*CNX*; GRMZM2G134668) (Fig. 6a). Proteins that enter the eukaryotic secretory pathway are modified and folded into their native structures within the endoplasmic reticulum. Protein folding is an active process that is assisted by catalysts and chaperones such as the immunoglobulin heavy chain-lumen binding protein (*BiP*), calreticulin, *CNX*, and *PDI* [57–59]. For example, one of the early events involved in the heat stress pathway that induces extensive downstream gene expression is the activation by the critical transcriptional regulators, namely the heat shock factors (HSFs) [60, 61]. The HSFs are evolutionarily conserved winged helix-turn-helix proteins that preferentially bind to *cis*-acting DNA promoter regions known as heat shock elements [62]. The HSFs are critical for the viability of various fungal species and control major developmental processes in higher eukaryotes, suggesting that they regulate basal transcription, in addition to functioning in stress responses [63–67]. The capacity of HSFs to respond to various cellular stresses is affected by the negative regulatory role of chaperones, modulation of nucleocytoplasmic shuttling, various post-translational modifications, and, in higher eukaryotes, the generation of trimers via aggregation of monomers [68]. *HSF* repression due to *Hsp90* and *Hsp70s-Hsp40* chaperone complexes [69–71] involves a negative feedback loop that titrates the production of chaperones, thereby facilitating optimal protein folding [72]. The higher *ZmCCT* expression levels in HZ4-NIL than in HZ4 under LD conditions observed in the present study resulted in the greater upregulation of *PDI*s, *BiP* (*Hsp70*), *Hsp40*, *CNX*, and *Hsp90* (*GRMZM2G024668*) in HZ4-NIL under LD conditions and during exposure to abiotic stress under LD conditions (Fig. 5b). This may explain why HZ4-NIL was more tolerant to abiotic stress than HZ4 (Additional file 3: Figure S1a and b). PDIs are the key protein folding catalysts that are activated during the unfolded protein response (UPR). Furthermore, the UPR induces the upregulation of *AtPDI* genes [73]. Hsp90 is an important stress response protein. When exposed to stress, Hsp90 stabilizes protein structures and membrane systems, which prevents the aggregation of proteins and enables the refolding of misfolded proteins [74]. In transgenic *A. thaliana*, the overexpression of *GmHsp90* decreases abiotic stress damage and maintains growth and development [75], while the expression of *Hsp70* and *Hsp40* enhances heat tolerance [76–78]. These results confirm that the upregulation of these genes leads to increased tolerance to abiotic stresses in plants.

The expression of *ZmCCA1* affected the expression of *BAKEKK1* (GRMZM2G328258), *FLS2* (GRMZM2G040508), and *MKK1/2* (GRMZM2G049695) (Fig. 5a). Pathogen-associated molecular pattern (PAMP)-triggered innate immunity is considered the first line of defense in plants. PAMP signals are perceived by highly specific receptors located in the plasma membrane, including the flagellin receptor *FLS2* [79]. *BAK1* interacts with *FLS2* upon binding of the ligand flg22, and is required for activating physiological responses [80]. Asai et al. [81] described a complete plant MAP kinase cascade (*MEKK1*, *MKK4/MKK5*, and *MPK3/MPK6*) and determined that *WRKY22/WRKY29* transcription factors influence downstream events involving *FLS2*. The activation of this MAPK cascade results in resistance to bacterial and fungal pathogens. The higher *ZmCCA1* expression levels in HZ4-NIL than in HZ4 under LD conditions observed in the present study led to greater expression of *BAKEKK1*, *FLS2*, and *MKK1/2* in HZ4-NIL under LD conditions (Fig. 5a). This resulted in HZ4-NIL being more tolerant to biotic stress than HZ4 (Additional file 3: Figure S1c). Consistent with these findings, *BAK1* contributes to the resistance of *A. thaliana* to infections by the hemibiotrophic bacterium *Pseudomonas syringae* or the obligate biotrophic oomycete *Hyaloperonospora arabidopsidis* [82]. The *FLS2* homologs in rice, tobacco, and tomato recognize flg22 as part of another type of resistance response, and are required for immunity against bacteria [83]. The *MEKK1-MKK1/MKK2-MPK4* cascade represses cell death and immune responses, whereas programmed cell death and defense responses are constitutively activated in *A. thaliana mekk1*, *mkk1 mkk2*, and *mpk4* mutants [84].

## Conclusions

We identified a set of genes with higher expression in HZ4-NIL than in HZ4 using RNA-seq. These genes function in circadian responses and some stress tolerance responses. Co-expression analysis and the diurnal rhythms of genes related to stress responses suggest that *ZmCCT* and one of the circadian clock core genes, *ZmCCA1*, are important nodes that link the photoperiod to stress tolerance responses under LD conditions.

## Additional files

Additional file 1: Table S1. Molecular markers developed for fine mapping of qDPS10 on chromosome 10. (XLSX 11 kb)
Additional file 2: Table S5. Primers used for validating differentially expressed genes and the identities of the co-expressed genes related to stress tolerance in HZ4 and HZ4-NIL. (XLSX 14 kb)
Additional file 3: Figure S1. Phenotypic responses to *Fusarium graminearum* and shoot apical meristem (SAM) morphologies of HZ4 and HZ4-NIL from the 3- to 7-fully expanded leaf stages under long-day conditions. (a) Phenotypes after artificial inoculation with *F. graminearum*. Red and blue arrows indicate HZ4 and HZ4-NIL plants, respectively. (b) SAM morphologies of HZ4 and HZ4-NIL plants in the 3- to 7-fully expanded leaf stages under long-day conditions. **Figure S2.** Summary of reads analysis. Results of Illumina transcriptome sequence data (a, b) and quality control (c, d) for leaves and shoot apices of HZ4 and HZ4-NIL. **Figure S3.** Sample clusters according to the gene expression profiles of HZ4 and HZ4-NIL leaves and shoot apices. **Figure S4.** Comparison of leaf and shoot apex gene expression patterns between HZ4 and HZ4-NIL. (a) Venn diagram of expressed genes identified in the leaves and shoot apices of HZ4 and HZ4-NIL. (b) Number of differentially expressed genes (DEGs) identified in HZ4 and HZ4-NIL in different developmental stages. (c) Venn diagram of up- and downregulated DEGs in the leaves and shoot apical meristems of HZ4-NIL relative to the levels in HZ4 at all leaf stages under long-day conditions. **Figure S5.** Gene Ontology classification of common differentially expressed genes (DEGs) in different organs. The DEGs are grouped under three hierarchically structured GO terms: biological process, cellular component, and molecular function. The y-axis indicates the number and percentage of proteins in each GO term. (PPTX 2130 kb)
Additional file 4:
**Table S6.** Molecular markers developed for fine mapping of qDPS10 on chromosome 10. (XLSX 358 kb)
Additional file 5: Table S2. Gene ontology enrichment of differentially expressed genes in HZ4 and HZ4-NIL in three development stages. (XLS 21 kb)
Additional file 6: Figure S6. Validation of the RNA-seq data for some differentially expressed genes identified in HZ4-NIL compared with HZ4 by qPCR. (DOCX 101 kb)
Additional file 7: Table S3. Gene symbols and genes co-expressed in HZ4 and HZ4-NIL. (XLSX 16 kb)
Additional file 8: Table S4.
*Cis*-acting regulatory elements identified in the promoter regions of genes in the nodes of the co-expression network. (XLSX 13 kb)

## Abbreviations

BiP: Immunoglobulin heavy chain-lumen binding protein
CCA1: CIRCADIAN CLOCK ASSOCIATED 1
CNX: Calnexin
conz1: CONSTANS 1
DLF1: DELAYED FLOWERING 1
FT: Flowering locus T
GI: GIGANTEA
Hd3a: HEADING DATE 3a
HSF: Heat shock factor
HZ4: Huangzao4
HZ4-NIL: Huangzao4 near-isogenic line
ID1: INDETERMINATE 1
LD: Long day
LHY: LATE ELONGATED HYPOCOTYL
PDI: Protein disulfide isomerase
qRT-PCR: quantitative reverse transcription polymerase chain reaction
RFT1: RICE FLOWERING LOCUS T1
TOC1: TIMING OF CAB EXPRESSION 1
UPR: Unfolded protein response
ZCN8: CENTRORADIALIS 8
ZmCCT-AQ: ZmCCT-associated QTL
