# Supplementary material for: Dual functions of the ZmCCT-associated quantitative trait locus in flowering and stress responses under long-day conditions
Source: BMC Plant Biol. 2016 Nov 3;16:239. doi: 10.1186/s12870-016-0930-1 (PMC5094027; doi:10.1186/s12870-016-0930-1)
Supplement: Additional file 3: Figure S1. — Phenotypic responses to Fusarium graminearum and shoot apical meristem (SAM) morphologies of HZ4 and HZ4-NIL from the 3- to 7-fully expanded leaf stages under long-day conditions. (a) Phenotypes after artificial inoculation with F. graminearum. Red and blue arrows indicate HZ4 and HZ4-NIL plants, respectively. (b) SAM morphologies of HZ4 and HZ4-NIL plants in the 3- to 7-fully expanded leaf stages under long-day conditions. Figure S2. Summary of reads analysis. Results of Illumina transcriptome sequence data (a, b) and quality control (c, d) for leaves and shoot apices of HZ4 and HZ4-NIL. Figure S3. Sample clusters according to the gene expression profiles of HZ4 and HZ4-NIL leaves and shoot apices. Figure S4. Comparison of leaf and shoot apex gene expression patterns between HZ4 and HZ4-NIL. (a) Venn diagram of expressed genes identified in the leaves and shoot apices of HZ4 and HZ4-NIL. (b) Number of differentially expressed genes (DEGs) identified in HZ4 and HZ4-NIL in different developmental stages. (c) Venn diagram of up- and downregulated DEGs in the leaves and shoot apical meristems of HZ4-NIL relative to the levels in HZ4 at all leaf stages under long-day conditions. Figure S5. Gene Ontology classification of common differentially expressed genes (DEGs) in different organs. The DEGs are grouped under three hierarchically structured GO terms: biological process, cellular component, and molecular function. The y-axis indicates the number and percentage of proteins in each GO term. (PPTX 2130 kb) [file 12870_2016_930_MOESM3_ESM.pptx]

## Slide 1
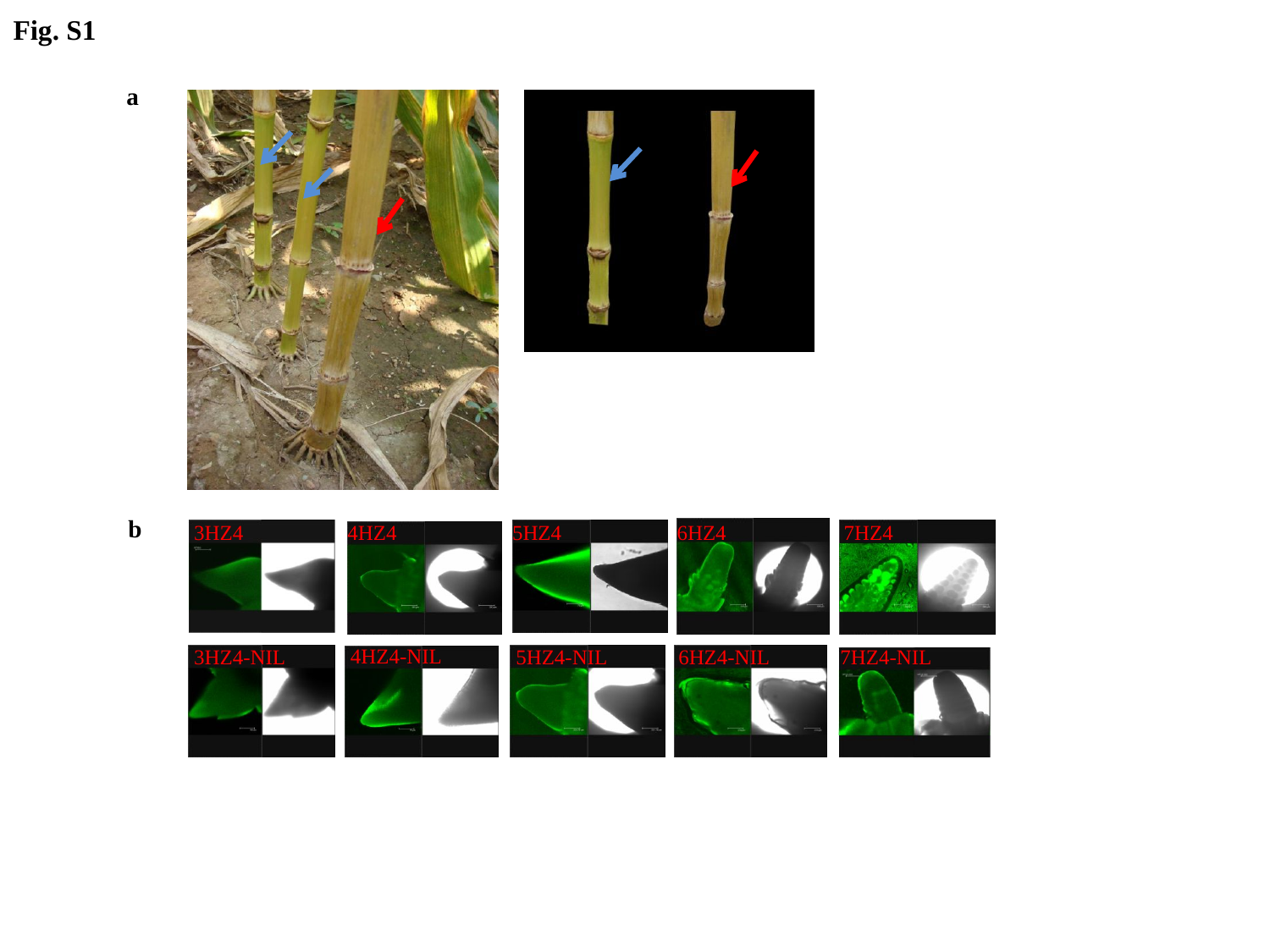

Fig. S1
a
b
3HZ4
4HZ4
5HZ4
6HZ4
7HZ4
4HZ4-NIL
6HZ4-NIL
3HZ4-NIL
5HZ4-NIL
7HZ4-NIL

## Slide 2
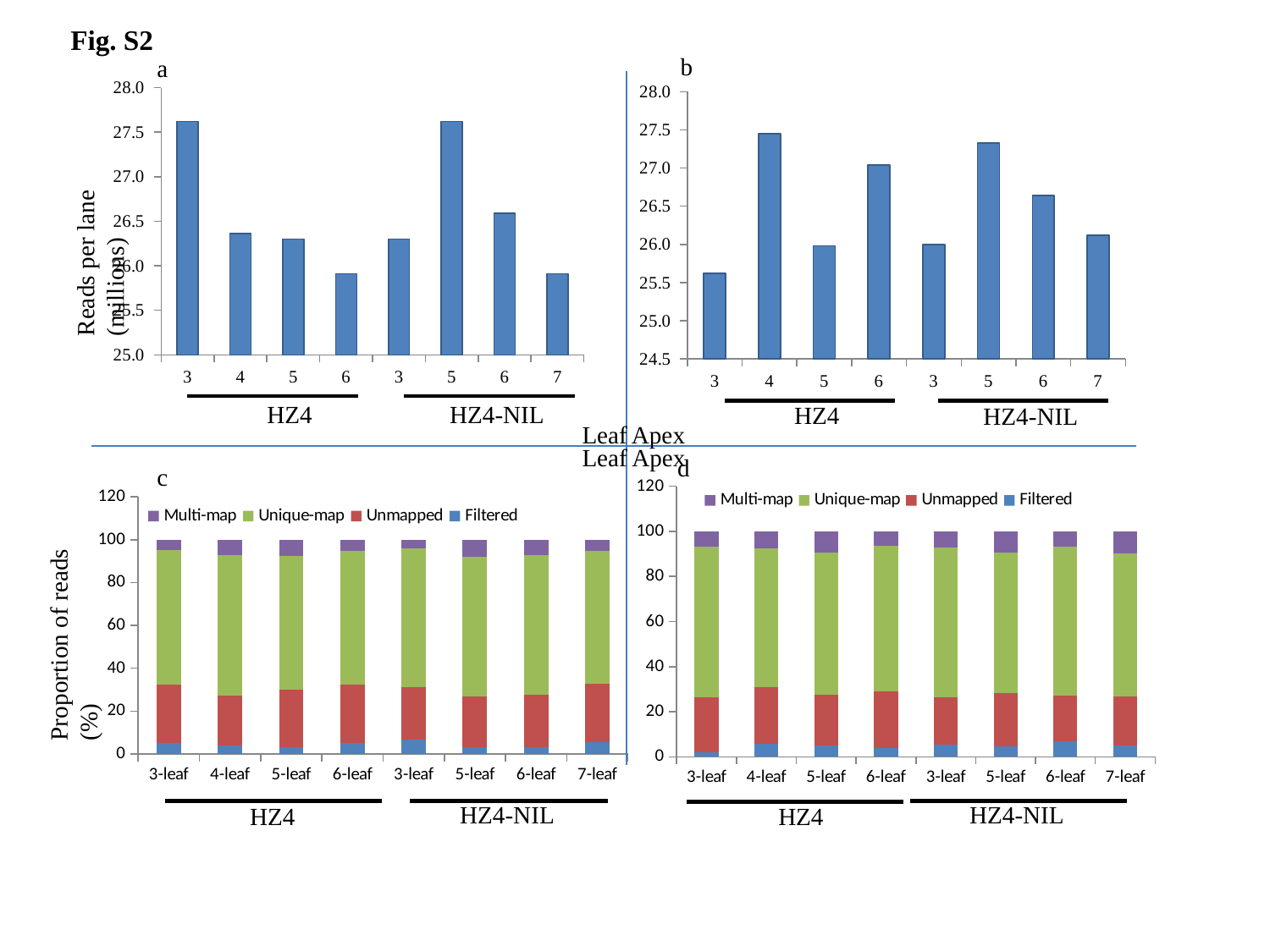

Fig. S2
b
a
### Chart
| Category | replicate I |
|---|---|
| 3 | 27.62 |
| 4 | 26.36 |
| 5 | 26.3 |
| 6 | 25.91 |
| 3 | 26.3 |
| 5 | 27.62 |
| 6 | 26.59 |
| 7 | 25.91 |
### Chart
| Category | replicate I |
|---|---|
| 3 | 25.62 |
| 4 | 27.45 |
| 5 | 25.979999999999997 |
| 6 | 27.04 |
| 3 | 26.0 |
| 5 | 27.330000000000002 |
| 6 | 26.64 |
| 7 | 26.12 |Reads per lane (millions)
HZ4
HZ4-NIL
HZ4
HZ4-NIL
Leaf
Apex
Leaf
Apex
c
HZ4-NIL
HZ4-NIL
HZ4
HZ4
Proportion of reads (%)
d
### Chart
| Category | Filtered | Unmapped | Unique-map | Multi-map |
|---|---|---|---|---|
| 3-leaf | 2.03 | 24.18 | 67.14 | 6.649999999999999 |
| 4-leaf | 5.55 | 25.18 | 61.849999999999994 | 7.42 |
| 5-leaf | 4.89 | 22.7 | 63.010000000000005 | 9.4 |
| 6-leaf | 4.03 | 25.05 | 64.69 | 6.23 |
| 3-leaf | 5.24 | 21.23 | 66.26 | 7.2700000000000005 |
| 5-leaf | 4.67 | 23.71 | 62.160000000000004 | 9.460000000000003 |
| 6-leaf | 6.8 | 20.29 | 66.28 | 6.63 |
| 7-leaf | 4.83 | 21.75 | 63.79000000000001 | 9.63 |
### Chart
| Category | Filtered | Unmapped | Unique-map | Multi-map |
|---|---|---|---|---|
| 3-leaf | 5.21 | 27.110000000000003 | 62.75 | 4.930000000000001 |
| 4-leaf | 3.86 | 23.49 | 65.33 | 7.3199999999999985 |
| 5-leaf | 3.2800000000000002 | 26.759999999999998 | 62.379999999999995 | 7.58 |
| 6-leaf | 5.3199999999999985 | 27.02 | 62.42 | 5.24 |
| 3-leaf | 6.619999999999998 | 24.459999999999997 | 64.78 | 4.14 |
| 5-leaf | 2.9899999999999998 | 24.02 | 65.16999999999999 | 7.8199999999999985 |
| 6-leaf | 3.3299999999999996 | 24.12 | 65.29 | 7.26 |
| 7-leaf | 5.45 | 27.32 | 61.97 | 5.26 |

## Slide 3
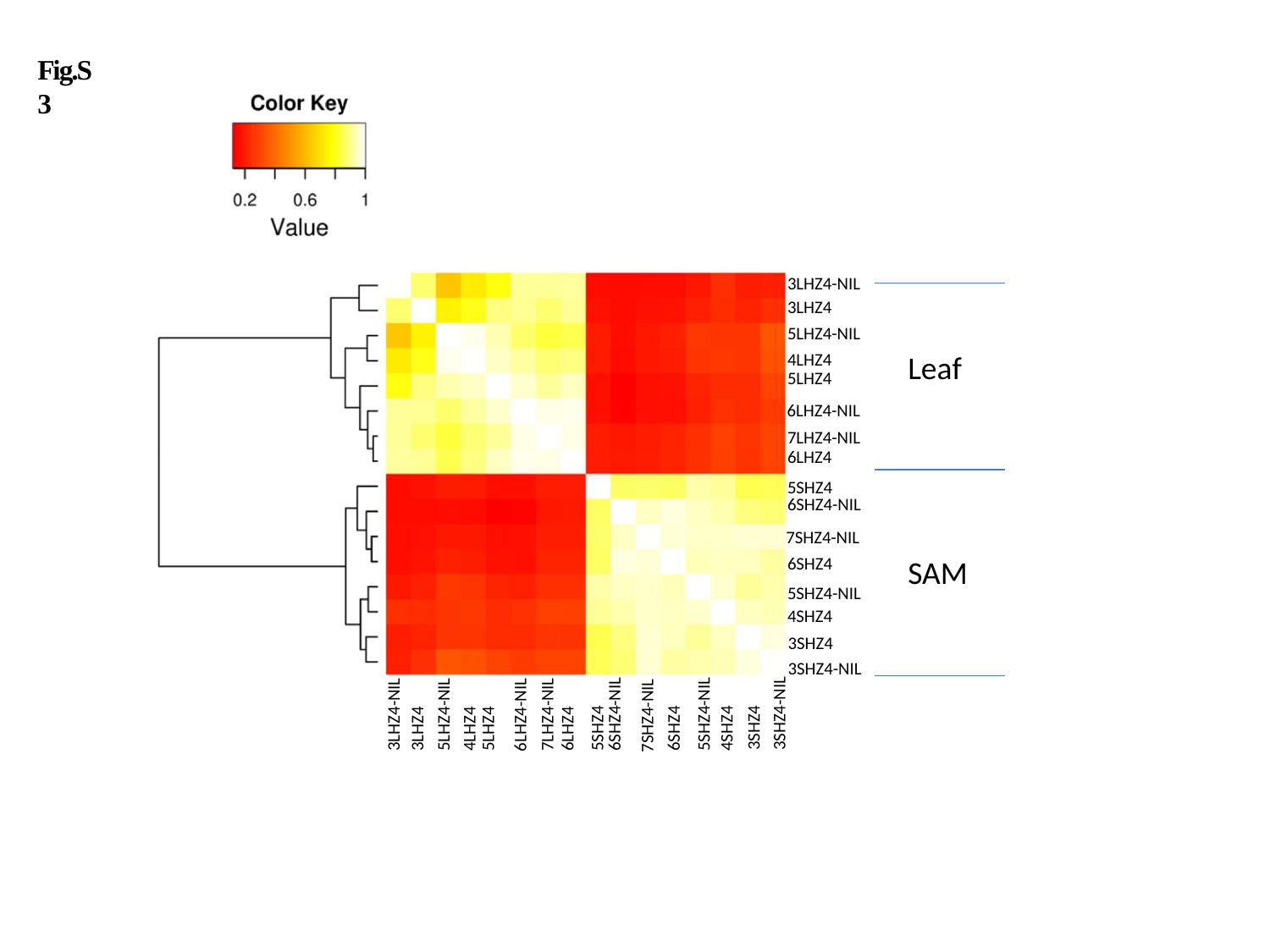

Fig.S3
3LHZ4-NIL
3LHZ4
5LHZ4-NIL
4LHZ4
5LHZ4
6LHZ4-NIL
7LHZ4-NIL
6LHZ4
5SHZ4
6SHZ4-NIL
7SHZ4-NIL
6SHZ4
5SHZ4-NIL
4SHZ4
3SHZ4
3SHZ4-NIL
Leaf
SAM
3SHZ4
3SHZ4-NIL
3LHZ4-NIL
3LHZ4
5LHZ4-NIL
4LHZ4
5LHZ4
7LHZ4-NIL
6LHZ4
5SHZ4
6SHZ4-NIL
6SHZ4
5SHZ4-NIL
4SHZ4
6LHZ4-NIL
7SHZ4-NIL

## Slide 4
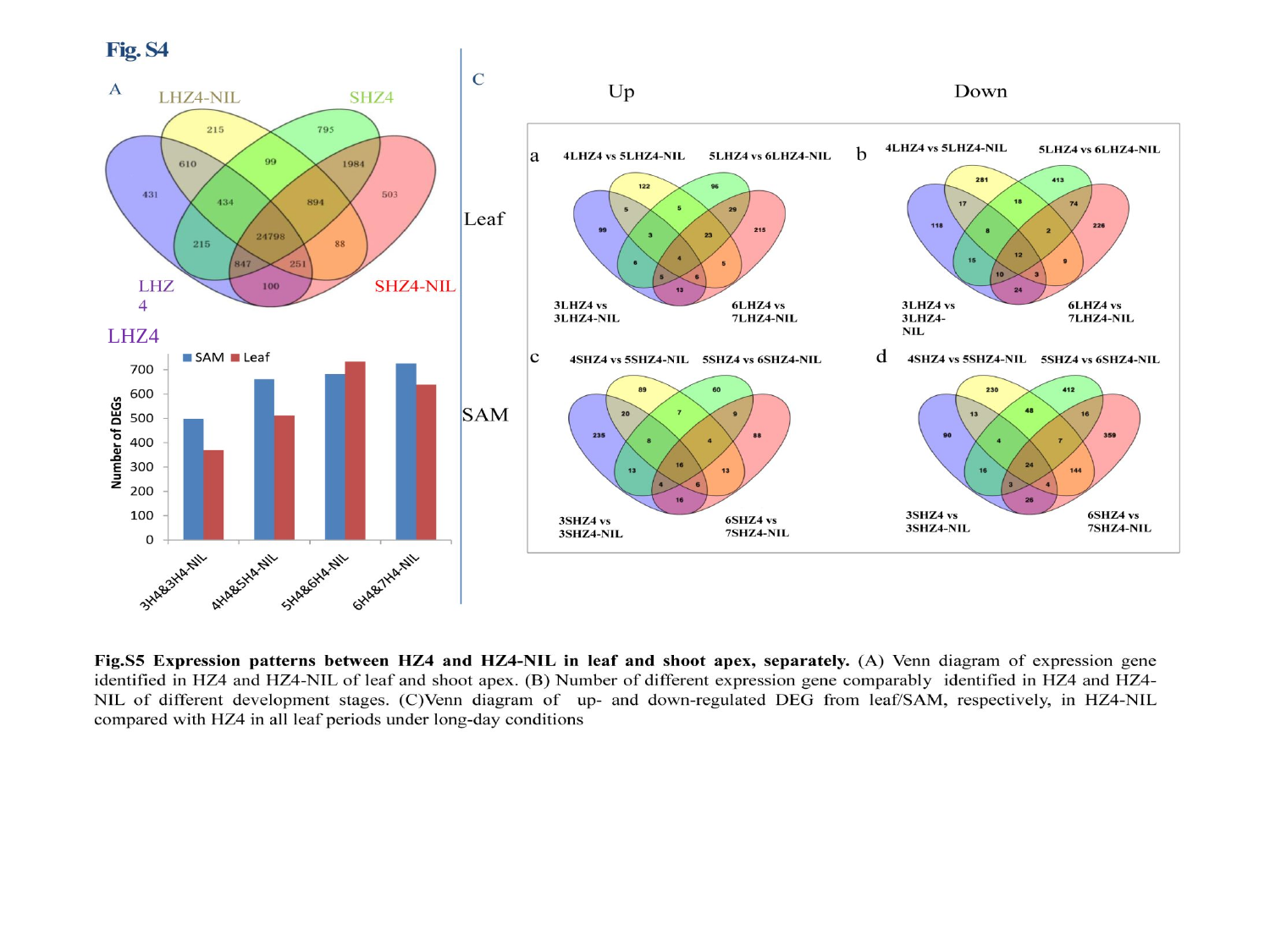

LHZ4

## Slide 5
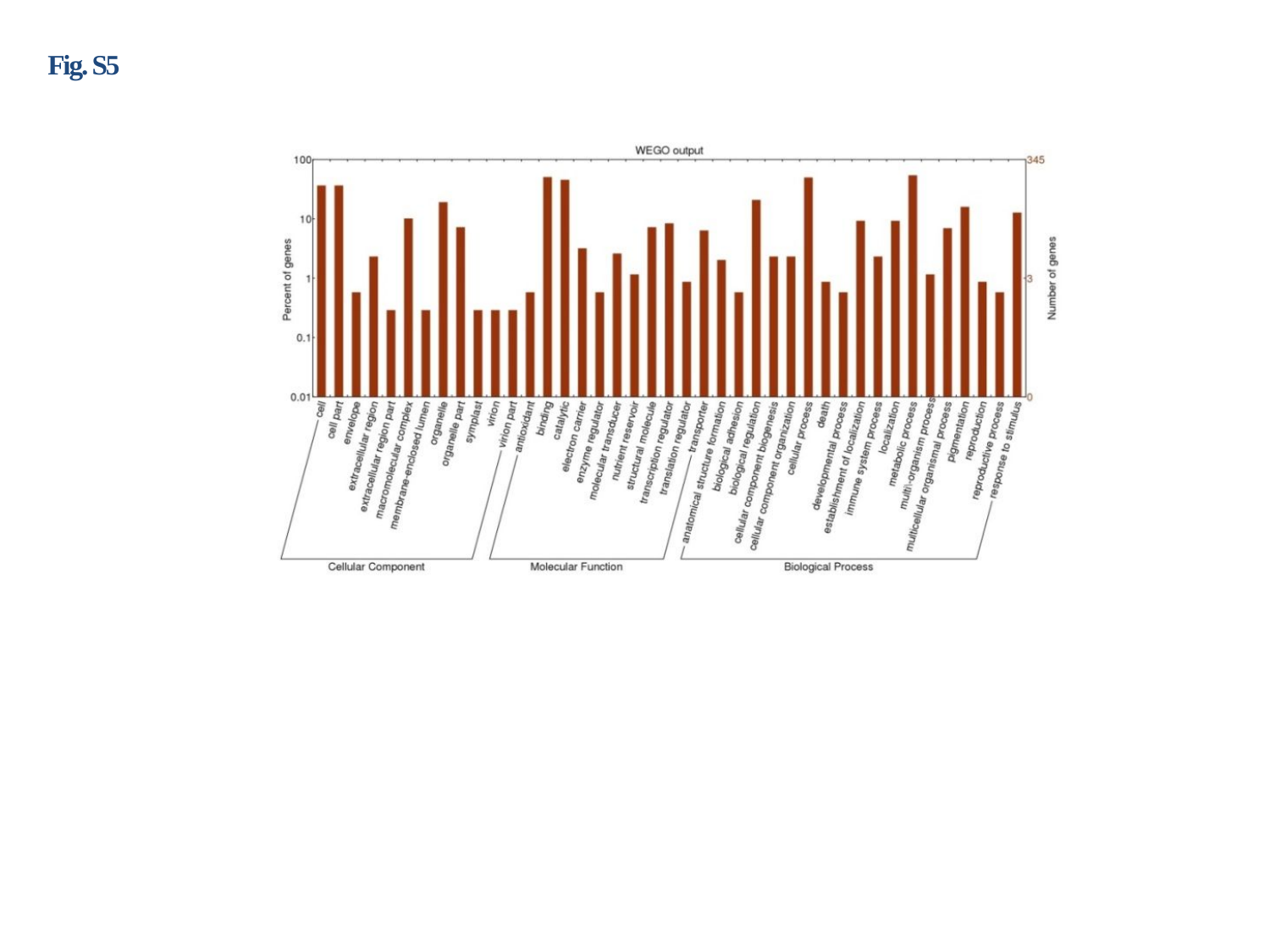

Fig. S5
