# Supplementary material for: Dual functions of the ZmCCT-associated quantitative trait locus in flowering and stress responses under long-day conditions
Source: BMC Plant Biol. 2016 Nov 3;16:239. doi: 10.1186/s12870-016-0930-1 (PMC5094027; doi:10.1186/s12870-016-0930-1)
Supplement: Supplementary file 4 [file 12870_2016_930_MOESM6_ESM.docx]

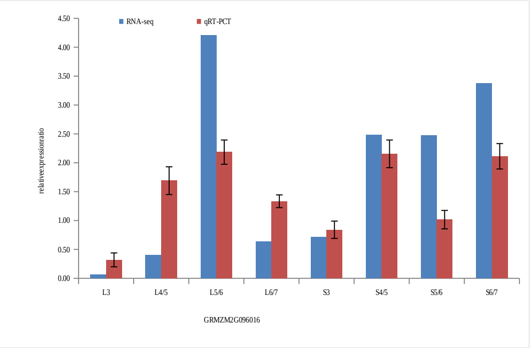
RNA-seq
qRT-PCT
relativeexpressionratio
L3 L4/5 L5/6 L6/7 S3 S4/5 S5/6 S6/7
GRMZM2G096016

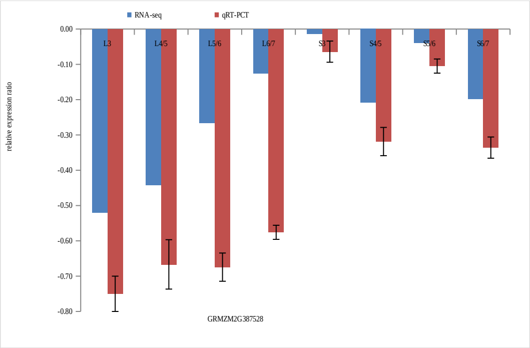
RNA-seq
qRT-PCT
relative expression ratio
L3 L4/5 L5/6 L6/7 S3 S4/5 S5/6 S6/7
GRMZM2G387528

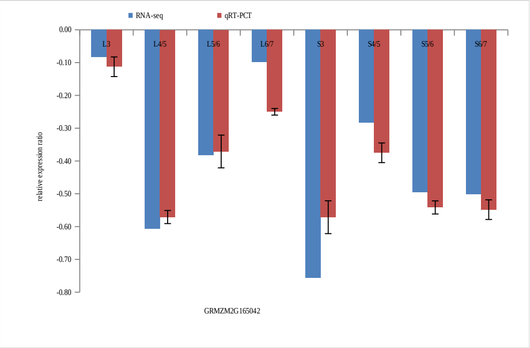
RNA-seq
qRT-PCT
relative expression ratio
L3 L4/5 L5/6 L6/7 S3 S4/5 S5/6 S6/7
GRMZM2G165042

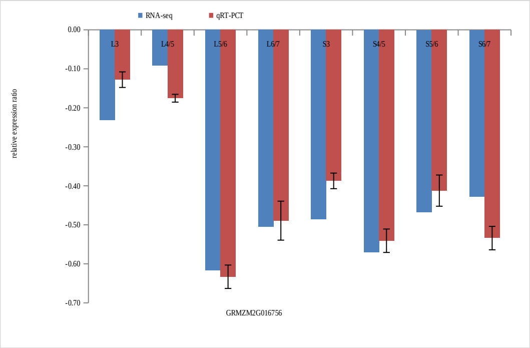
RNA-seq
qRT-PCT
relative expression ratio
L3 L4/5 L5/6 L6/7 S3 S4/5 S5/6 S6/7
GRMZM2G016756

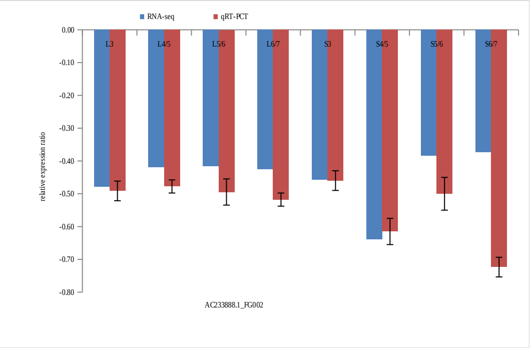
RNA-seq
qRT-PCT
relative expression ratio
L3 L4/5 L5/6 L6/7 S3 S4/5 S5/6 S6/7
AC233888.1_FG002

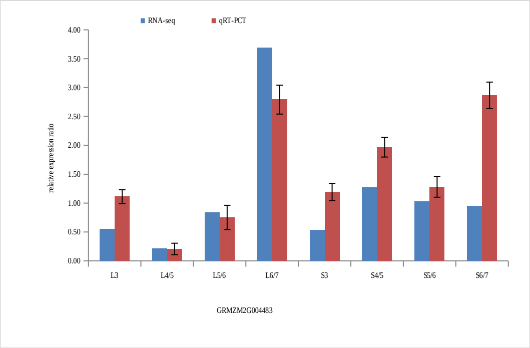
RNA-seq
qRT-PCT
relative expression ratio
L3 L4/5 L5/6 L6/7 S3 S4/5 S5/6 S6/7
GRMZM2G004483

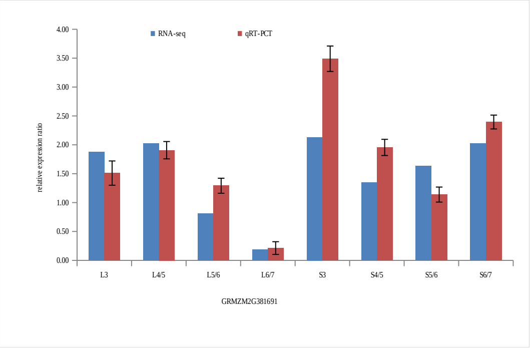
RNA-seq
qRT-PCT
relative expression ratio
L3 L4/5 L5/6 L6/7 S3 S4/5 S5/6 S6/7
GRMZM2G381691

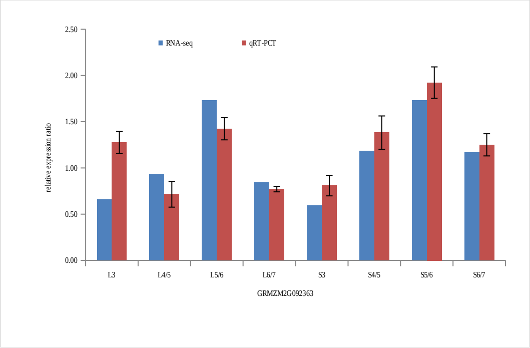
RNA-seq
qRT-PCT
relative expression ratio
L3 L4/5 L5/6 L6/7 S3 S4/5 S5/6 S6/7
GRMZM2G092363

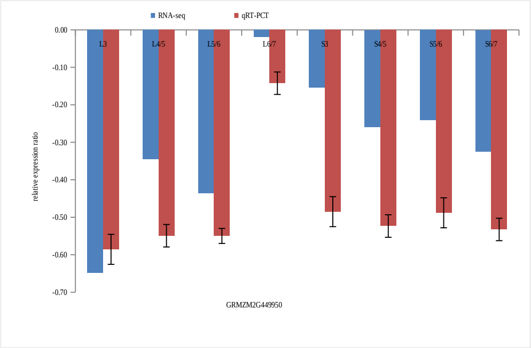
RNA-seq
qRT-PCT
relative expression ratio
L3
L4/5
L5/6
L6/7
S3
S4/5
S5/6
S6/7
GRMZM2G449950

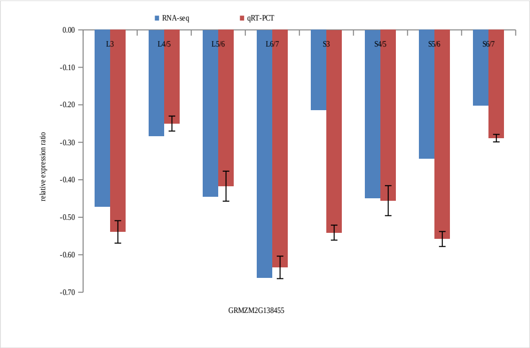
RNA-seq
qRT-PCT
relative expression ratio
L3
L4/5
L5/6
L6/7
S3
S4/5
S5/6
S6/7
GRMZM2G138455

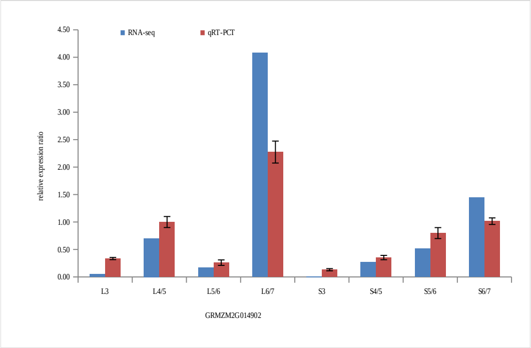
RNA-seq
qRT-PCT
relative expression ratio
L3
L4/5
L5/6
L6/7
S3
S4/5
S5/6
S6/7
GRMZM2G014902

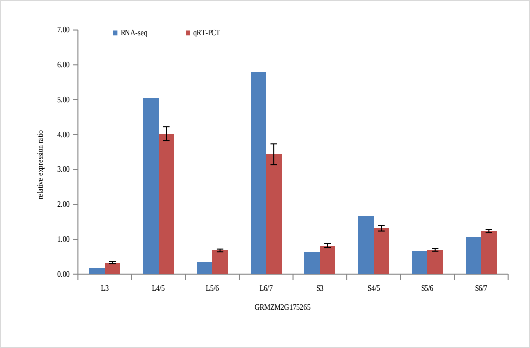
RNA-seq
qRT-PCT
relative expression ratio
L3
L4/5
L5/6
L6/7
S3
S4/5
S5/6
S6/7
GRMZM2G175265

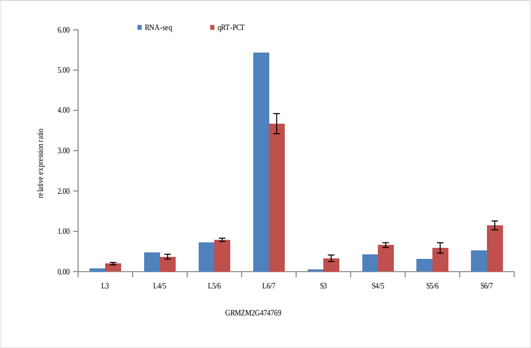
RNA-seq
qRT-PCT
relative expression ratio
L3
L4/5
L5/6
L6/7
S3
S4/5
S5/6
S6/7
GRMZM2G474769

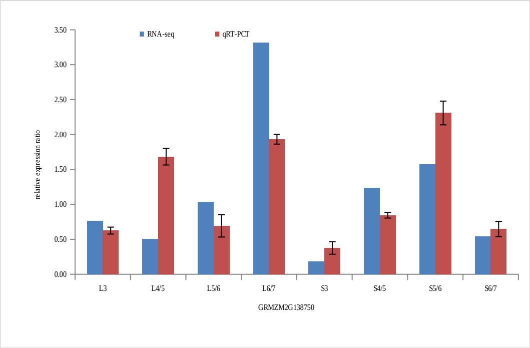
RNA-seq
qRT-PCT
relative expression ratio
L3
L4/5
L5/6
L6/7
S3
S4/5
S5/6
S6/7
GRMZM2G138750

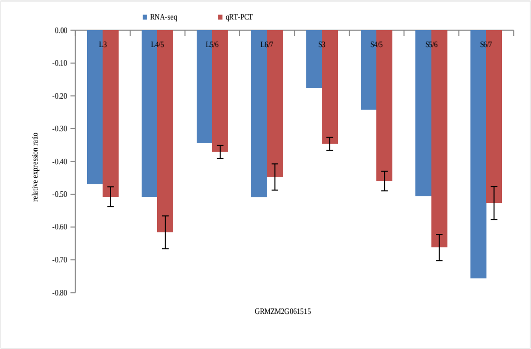
RNA-seq
qRT-PCT
relative expression ratio
L3
L4/5
L5/6
L6/7
S3
S4/5
S5/6
S6/7
GRMZM2G061515

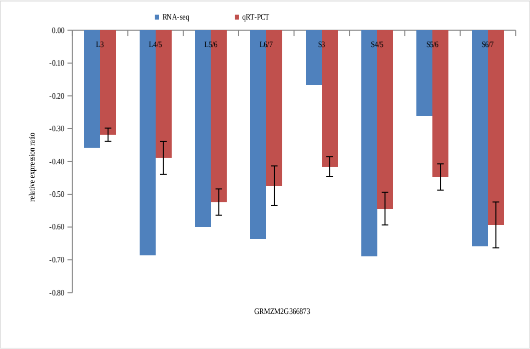
RNA-seq
qRT-PCT
relative expression ratio
L3
L4/5
L5/6
L6/7
S3
S4/5
S5/6
S6/7
GRMZM2G366873

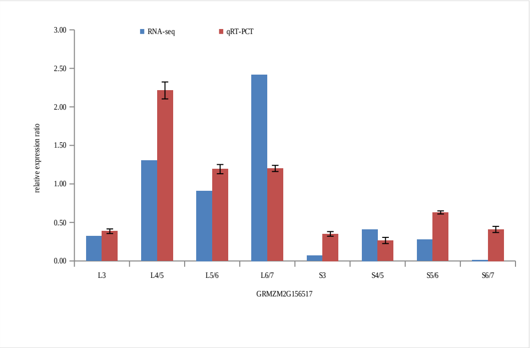
RNA-seq
qRT-PCT
relative expression ratio
3.00
2.50
2.00
1.50
1.00
0.50
0.00
L3
L4/5
L5/6
L6/7
S3
S4/5
S5/6
S6/7
GRMZM2G156517

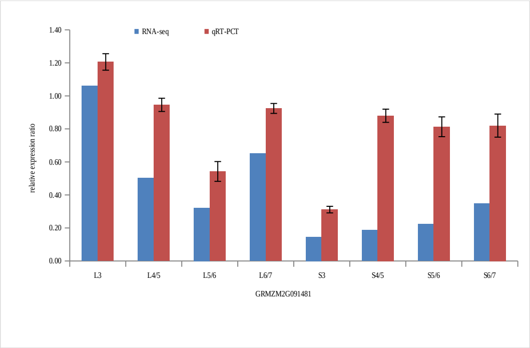
RNA-seq
qRT-PCT
relative expression ratio
1.40
1.20
1.00
0.80
0.60
0.40
0.20
0.00
L3
L4/5
L5/6
L6/7
S3
S4/5
S5/6
S6/7
GRMZM2G091481

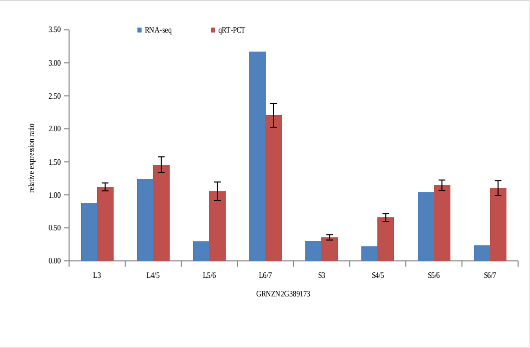
RNA-seq
qRT-PCT
relative expression ratio
3.50
3.00
2.50
2.00
1.50
1.00
0.50
0.00
L3
L4/5
L5/6
L6/7
S3
S4/5
S5/6
S6/7
GRNZN2G389173

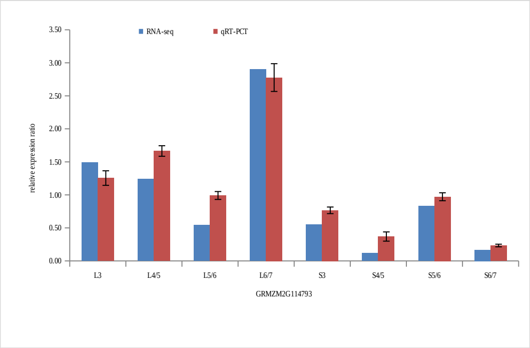
RNA-seq
qRT-PCT
relative expression ratio
3.50
3.00
2.50
2.00
1.50
1.00
0.50
0.00
L3
L4/5
L5/6
L6/7
S3
S4/5
S5/6
S6/7
GRMZM2G114793

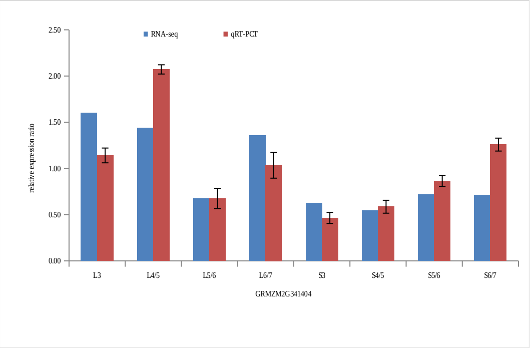
RNA-seq
qRT-PCT
relative expression ratio
2.50
2.00
1.50
1.00
0.50
0.00
L3
L4/5
L5/6
L6/7
S3
S4/5
S5/6
S6/7
GRMZM2G341404

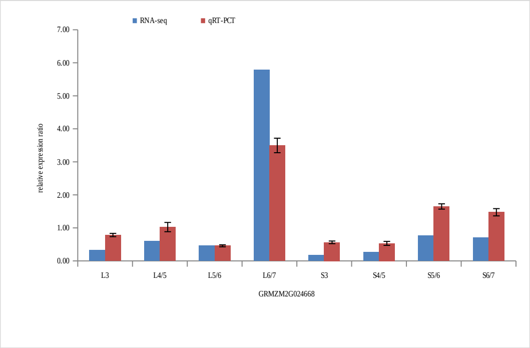
RNA-seq
qRT-PCT
relative expression ratio
7.00
6.00
5.00
4.00
3.00
2.00
1.00
0.00
L3
L4/5
L5/6
L6/7
S3
S4/5
S5/6
S6/7
GRMZM2G024668

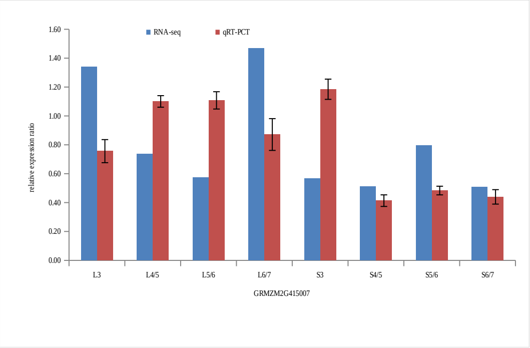
RNA-seq
qRT-PCT
relative expression ratio
1.60
1.40
1.20
1.00
0.80
0.60
0.40
0.20
0.00
L3
L4/5
L5/6
L6/7
S3
S4/5
S5/6
S6/7
GRMZM2G415007

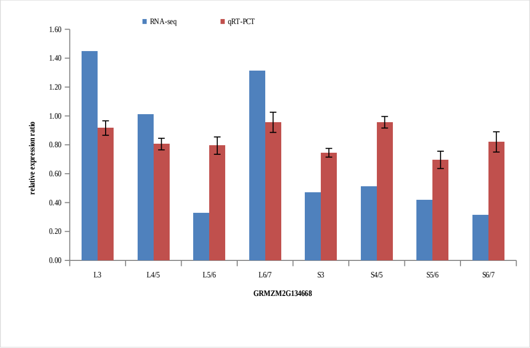
RNA-seq
qRT-PCT
relative expression ratio
1.60
1.40
1.20
1.00
0.80
0.60
0.40
0.20
0.00
L3
L4/5
L5/6
L6/7
S3
S4/5
S5/6
S6/7
GRMZM2G134668

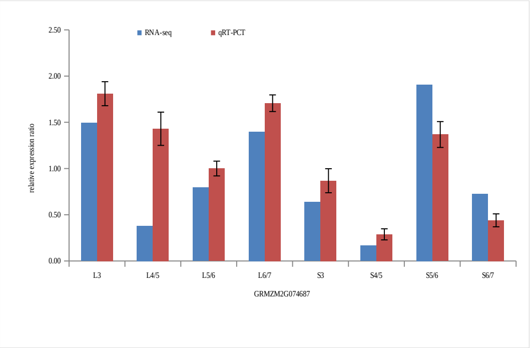
RNA-seq
qRT-PCT
relative expression ratio
0.00
0.50
1.00
1.50
2.00
2.50
L3
L4/5
L5/6
L6/7
S3
S4/5
S5/6
S6/7
GRMZM2G074687

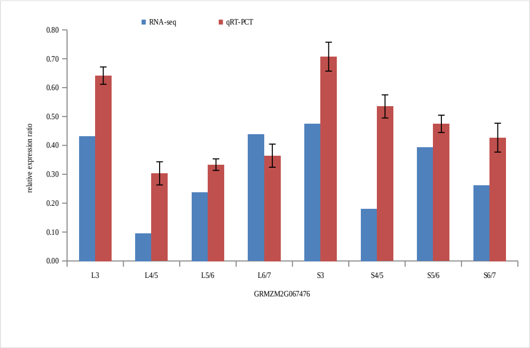
RNA-seq
qRT-PCT
relative expression ratio
0.00
0.10
0.20
0.30
0.40
0.50
0.60
0.70
0.80
L3
L4/5
L5/6
L6/7
S3
S4/5
S5/6
S6/7
GRMZM2G067476

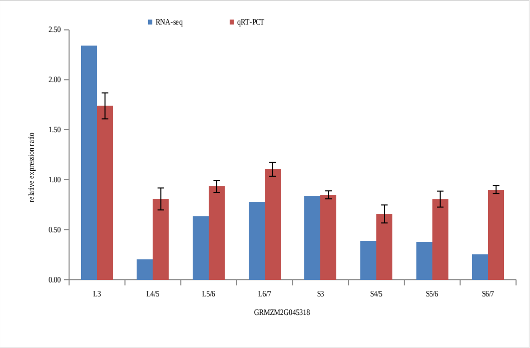
RNA-seq
qRT-PCT
relative expression ratio
0.00
0.50
1.00
1.50
2.00
2.50
L3
L4/5
L5/6
L6/7
S3
S4/5
S5/6
S6/7
GRMZM2G045318

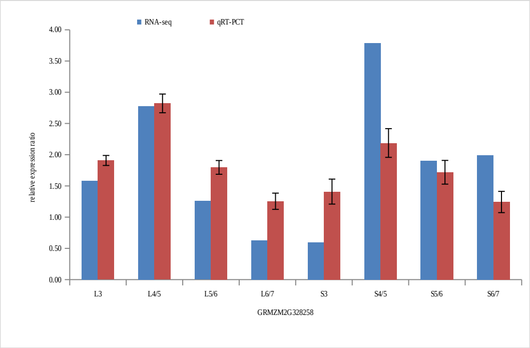
RNA-seq
qRT-PCT
relative expression ratio
0.00
0.50
1.00
1.50
2.00
2.50
3.00
3.50
4.00
L3
L4/5
L5/6
L6/7
S3
S4/5
S5/6
S6/7
GRMZM2G328258

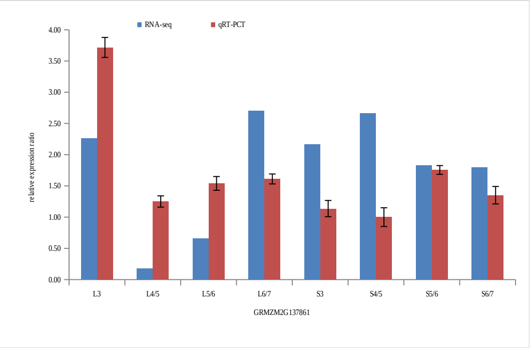
RNA-seq
qRT-PCT
relative expression ratio
0.00
0.50
1.00
1.50
2.00
2.50
3.00
3.50
4.00
L3
L4/5
L5/6
L6/7
S3
S4/5
S5/6
S6/7
GRMZM2G137861

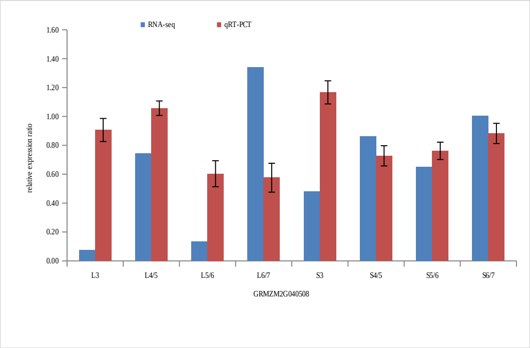
RNA-seq
qRT-PCT
relative expression ratio
0.00
0.20
0.40
0.60
0.80
1.00
1.20
1.40
1.60
L3
L4/5
L5/6
L6/7
S3
S4/5
S5/6
S6/7
GRMZM2G040508

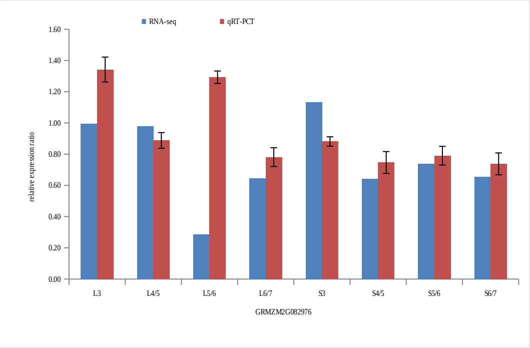
RNA-seq
qRT-PCT
relative expression ratio
0.00
0.20
0.40
0.60
0.80
1.00
1.20
1.40
1.60
L3
L4/5
L5/6
L6/7
S3
S4/5
S5/6
S6/7
GRMZM2G082976

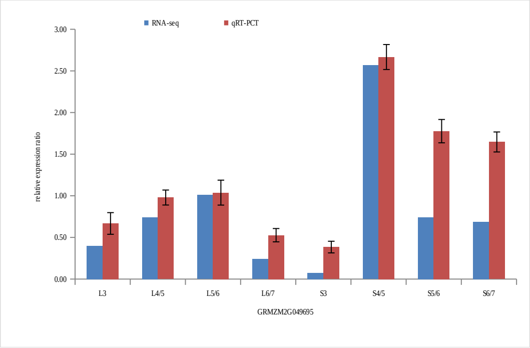
RNA-seq
qRT-PCT
relative expression ratio
0.00
0.50
1.00
1.50
2.00
2.50
3.00
L3
L4/5
L5/6
L6/7
S3
S4/5
S5/6
S6/7
GRMZM2G049695

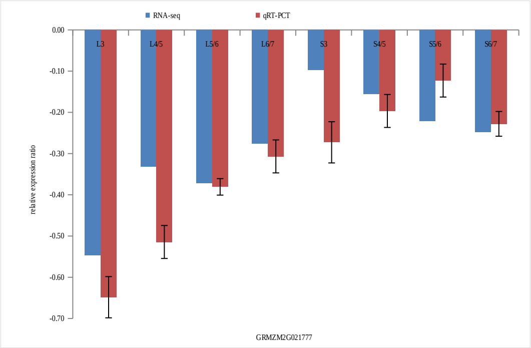
RNA-seq
qRT-PCT
0.00
-0.10
-0.20
-0.30
-0.40
-0.50
-0.60
-0.70
relative expression ratio
L3
L4/5
L5/6
L6/7
S3
S4/5
S5/6
S6/7
GRMZM2G021777
